# Supplementation With Curcumin Exhibits Nephroprotective Effects in Cisplatin‐Induced Acute Kidney Injury

**DOI:** 10.1002/fsn3.71240

**Published:** 2025-11-28

**Authors:** Mingkang Zhang, Yazhi Wang, Xiujuan Wang, Yan Zhou, Yanrong Ma, Xin'an Wu

**Affiliations:** ^1^ School of Pharmacy Lanzhou University Lanzhou China; ^2^ Department of Pharmacy The First Hospital of Lanzhou University Lanzhou China; ^3^ Engineering Research Centre of Prevention and Control for Clinical Medication Risk Lanzhou China; ^4^ The Second School of Clinical Medicine Lanzhou University Lanzhou China

**Keywords:** AMPK/PGC‐1α/Sirt3 pathway, Bax/Bcl2/Caspase‐3 pathway, cisplatin, curcumin supplementation, nephrotoxicity, Nrf2/Keap1 pathway

## Abstract

Cisplatin is used as a broad‐spectrum chemotherapeutic agent for the treatment of many solid malignancies. With the widespread use of cisplatin, the side effects (especially nephrotoxicity) exhibited are becoming increasingly evident. There are no clinically effective drugs to prevent or treat cisplatin‐induced nephrotoxicity. Recently, curcumin has received widespread attention in the field of dietary supplements. In this study, we investigated whether curcumin could attenuate cisplatin‐induced renal tubular epithelial cell injury, mitochondrial damage, and reactive oxygen species (ROS) accumulation by in vitro experiments. Subsequently, we evaluated the effects of curcumin supplementation on mitochondrial dynamics, oxidative stress levels, and apoptosis induced by cisplatin. Moreover, renal transporter expression and urinary toxins accumulation were also assessed. Cisplatin‐induced nephrotoxicity was caused by dysregulation of mitochondrial homeostasis, leading to excessive oxidative stress and apoptosis in the kidney, as well as dysregulation of renal transporter expression, leading to the accumulation of urinary toxins. However, curcumin could restore mitochondrial homeostasis through the AMPK/PGC‐1α/Sirt3 pathway, alleviate renal oxidative stress and apoptosis through the Nrf2/Keap1 and Bax/Bcl2/Caspase‐3 pathways, and regulate renal transporter expression to alleviate urinary toxins accumulation, which ultimately alleviated cisplatin‐induced nephrotoxicity.

## Graphical abstract

(By Figdraw, ID: APASW31e60) (37.7 × 60 Mm). This study demonstrates that supplementation with curcumin alleviates cisplatin‐induced kidney injury. It works by restoring mitochondrial balance via the AMPK/PGC‐1α/Sirt3 pathway, reducing oxidative stress and apoptosis through the Nrf2/Keap1 and Bax/Bcl2/Caspase‐3 pathways, and correcting dysfunction in renal transporters. Thus, supplementation with curcumin exhibits nephroprotective effects in cisplatin‐induced acute kidney injury.

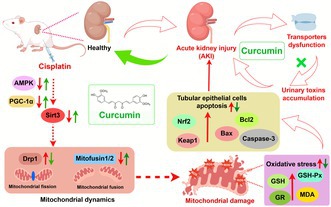

## 1 Introduction

Acute kidney injury (AKI) has developed into a global public health problem with high morbidity and mortality worldwide (Sabra et al. 2024). It has been reported that approximately 13 million people worldwide are diagnosed with AKI each year, and of these, about 1.7 million die (Huang et al. 2022). AKI is typically characterized clinically by a sudden loss of renal function over a short period (Wang et al. 2024). Although many clinical measures have been taken to prevent or treat the progression of AKI, some patients will eventually develop chronic kidney disease (CKD) or end‐stage renal disease (ESRD) (Huang et al. 2022). There are currently no effective therapeutic drugs for AKI in the clinic. Therefore, there is an urgent need for novel therapeutic approaches that can effectively alleviate AKI (Zou et al. 2021).

As the significant excretory organs in the body, the kidneys are more susceptible to chemical and drug toxicity (He et al. 2022). Cisplatin was the first broad‐spectrum platinum‐based antitumor chemotherapeutic agent approved by the US Food and Drug Administration (FDA) in 1978 (Zhang, Cui, et al. 2023). Owing to its remarkable therapeutic efficacy, it is commonly used in treating a variety of solid malignancies, such as ovarian, testicular, bladder, cervical, head and neck, and lung cancers (Shao et al. 2023). Notably, it has been reported that approximately 30% of patients treated with cisplatin will experience AKI (Zhang, Luo, et al. 2023). Furthermore, the therapeutic efficacy and nephrotoxicity of cisplatin exhibit a dose dependency (Fukushima et al. 2022).

In the body, cisplatin accumulates in renal tubules and causes AKI through various mechanisms, including DNA damage, oxidative stress, endoplasmic reticulum stress, inflammation, apoptosis, and necrotic apoptosis (Wang et al. 2022). Previous studies have suggested that mitochondria are the main target of cisplatin‐induced nephrotoxicity. It was reported that positively charged metabolites from hydrolysis of cisplatin accumulate in negatively charged mitochondria. Because of their high energy metabolism and density of mitochondria, renal proximal tubule cells are the most sensitive to the toxigenic effects of cisplatin (Jun et al. 2023). Moreover, cisplatin‐induced mitochondrial damage also increases the production of reactive oxygen species (ROS), triggers oxidative stress, and accelerates mitochondrial damage (Zhou, Dai, et al. 2022). The mechanism of cisplatin‐induced nephrotoxicity has not been fully clarified, and exploring the molecular mechanism of cisplatin nephrotoxicity has never ceased.

There are no clinically effective drugs to prevent or treat cisplatin‐induced nephrotoxicity. Numerous studies (including ours) are devoted to the search for highly effective, low‐toxicity drugs derived from natural products to prevent cisplatin‐induced AKI (Chun‐Yan et al. 2021). Notably, the curcumin obtained from the rhizomes of turmeric and other plants of the ginger family was once considered one of the most promising modern natural compounds to be developed (Jing et al. 2023). Extensive clinical trials have demonstrated that curcumin exhibits safety, efficacy, and minimal toxicity in human diseases (Subash C et al. 2012), and curcumin has received considerable attention as a dietary supplement (Siyu et al. 2022). In recent years, increasing evidence has reported that curcumin is used for hepatoprotective, hypoglycaemic, antitumor, antiproliferative, thromboprotective, neuroprotective, cardioprotective, and anti‐arthritic effects (Jianan et al. 2024; Ramadan et al. 2025). Moreover, other studies also reported that curcumin exhibited beneficial effects on AKI induced by rhabdomyolysis, gentamicin, ischemia–reperfusion, sepsis, potassium dichromate, and diclofenac sodium (Ahmady Y et al. 2017; Jindao et al. 2017). Numerous studies have demonstrated that curcumin exhibits powerful anti‐inflammatory and antioxidant properties and could effectively scavenge free radicals (Abd El‐Kader and Taha 2020; Malihe et al. 2022). Recent studies have suggested that curcumin attenuates renal injury induced by gentamicin (Jun et al. 2023) and potassium dichromate (Sabino Hazael et al. 2020) by regulating mitochondrial homeostasis, and preventing mitochondrial dysfunction and apoptosis. Curcumin may also be an alternative therapy for other diseases characterized by mitochondrial dysfunction (Zou et al. 2021). For example, Dongyao et al. reported that curcumin ameliorated sepsis‐induced cardiac dysfunction by reducing mitochondrial fission and promoting mitochondrial biogenesis (Dongyao et al. 2024). It is still unclear whether curcumin attenuates cisplatin‐induced AKI by reducing mitochondrial dysfunction, given that the mitochondria are the primary target of cisplatin‐induced nephrotoxicity.

It is possible to maintain renal function and overall health with a balanced diet and supplements when necessary, which contributes to curbing the risk factors for kidney injury (Cosola et al. 2018). This study investigated whether curcumin alleviated cisplatin‐induced renal tubular epithelial cell damage, mitochondrial impairment, and ROS accumulation via in vitro experiments. Subsequently, we evaluated the effects of curcumin supplementation on mitochondrial dynamics, oxidative stress levels, and apoptosis induced by cisplatin. Moreover, renal transporter expression and urinary toxin accumulation were also assessed.

## 2 Materials and Methods

### 2.1 Reagents and Materials

Cisplatin injection was purchased from Jiangsu Hansen Pharmaceutical Group Co. Ltd. (Lianyungang, Jiangsu, China). Curcumin (purity ≥ 98%), high‐efficiency RIPA lysis buffer, sodium carboxymethyl cellulose (CMC‐Na), SDS‐PAGE gel preparation kits, phenylmethylsulfonyl fluoride (PMSF), and Masson's trichrome staining kit were obtained from Solarbio Science and Technology Co. Ltd. (Beijing, China). Hematoxylin and eosin (HE) staining kit was provided by Servicebio Technology Co. Ltd. (Wuhan, Hubei, China). Physiological saline (0.9% NaCl) was purchased from Otsuka Pharmaceutical Co. Ltd. (Guangdong, China). Polyvinylidene difluoride (PVDF) membrane (0.45 μm pore size) was acquired from Merck Millipore Ltd. (Billerica, MA, USA). All other general‐use chemicals and solvents were obtained from Sigma‐Aldrich Corporation (St. Louis, MO, USA).

### 2.2 Cell Culture and Cell Viability Assays

Human renal proximal tubular epithelial cells (HK‐2) were cultured in MEM medium containing 10% fetal bovine serum and 1% (V/V) penicillin–streptomycin (PS; Gibco, USA). The environment of the cell culture incubator was maintained at 37°C and 5% CO_2_. To carry out the experiments, HK‐2 cells were seeded in a 96‐well plate at a density of 3 × 10^4^ to 5 × 10^4^ cells/mL for 24 h. After the cells adhered to the wall, they were cultured for 24 h in a fresh medium containing different concentrations of curcumin solution or a fresh medium containing cisplatin and curcumin, respectively. After reaching the set time, the Cell Counting Kit‐8 (CCK‐8) reagent (10 μL/well) was added. Then, the 96‐well plate was incubated at 37°C to develop the color. The absorbance of each well was measured at 450 nm (Ling et al. 2021). Cell viability was calculated as the ratio of treated wells' absorbance to untreated control wells. The wells containing PBS were used for calibration.

### 2.3 Reactive Oxygen Species (ROS) Detection

HK‐2 cells were inoculated into 20 mm glass‐bottomed cell culture dishes at a density of 3 to 5 × 10^4^ 10^4 cells/mL. The following day, once the cells were attached to the wall, they were incubated for 24 h with fresh serum‐free medium containing various concentrations of curcumin and 20 μM cisplatin. The cellular reactive oxygen species (ROS) content was detected according to the protocol described by Dongyao et al. (2024). In brief, the cells were incubated with serum‐free medium containing 10 μM DCFH‐DA for 20 min at 37°C in a cell culture incubator. The serum‐free medium removed the residual DCFH‐DA that had not wholly entered the cells. The level of ROS was detected using a fluorescence microscope (excitation wavelength was set at 488 nm, and the emission wavelength was set at 525 nm).

### 2.4 Mitochondrial Morphology Assessment

HK‐2 cells were inoculated into 20 mm glass‐bottomed cell culture dishes at a density of 3 to 5 × 10^4^ 10^4 cells/mL. The following day, once the cells were attached to the wall, they were incubated for 24 h with fresh serum‐free medium containing various concentrations of curcumin and 20 μM cisplatin. The mitochondrial morphology was assessed according to the previously described protocol (Li et al. 2023). In brief, the culture medium was discarded, and mitochondrial Red CMXRos Staining Solution pre‐warmed at 37°C was added. Incubate the cells at 37°C for 30 min. When the staining has finished, the medium is replaced with fresh serum‐free medium. The morphology of mitochondria was examined using a fluorescence microscope (excitation wavelength set to 579 nm, emission wavelength set to 599 nm).

### 2.5 Assessment of Mitochondrial Membrane Potential (MMP)

Mitochondrial membrane potential (MMP) was assessed according to the operation of Peerapanyasut et al. (2020). Briefly, once HK‐2 cells were grown to the appropriate density in 20 mm glass‐bottomed cell culture dishes, they were incubated with fresh serum‐free medium containing various concentrations of curcumin and 20 μM cisplatin for 24 h. Then, the medium was replaced with a medium containing JC‐1 staining working solution and incubated in a cell culture incubator at 37°C for 20 min. Then, the supernatant was aspirated and washed twice with JC‐1 staining buffer, and the serum‐free medium was added. The results were observed under a fluorescence microscope (the excitation wavelength was set at 585 nm and the emission wavelength was set at 590 nm for JC‐1 aggregates, and the excitation wavelength was set at 515 nm and the emission wavelength was set at 529 nm for JC‐1 monomers).

### 2.6 Animal Treatment

The animals used in the experiments were purchased from Lanzhou University Animal Experiment Centre. In which 8‐week‐old male Sprague–Dawley (SD) rats weighing 180–220 g were used in this experiment. The animal production license number is SCXK (Gan) 2020–0002. We housed all animals in the SPF Animal Experimentation Centre of Lanzhou University. We maintained the housing conditions at a temperature of 24°C, humidity of 40%, and a light–dark cycle of 12 h. The operations involved in this animal experiment were conducted under the guidance of the Animal Ethics Committee of the First Affiliated Hospital of Lanzhou University (Ethics Approval No. LDYYLL2021‐294). Furthermore, we ensured that the operations involved in the experimental animal experiments were performed under conditions that minimized animal suffering. All animal experiments followed the Animals (Scientific Procedures) Act 1986 and related guidelines in the UK, the European Union Directive 2010/63/EU on animal experimentation, and the National Research Council Guide for the Care and Use of Laboratory Animals. Moreover, all procedures involving animals in the study were under the ARRIVE guidelines.

### 2.7 Experimental Design

All animals were given a one‐week environmental acclimatization period before experiments were performed, and all animals had free access to food and purified sterile water during the experimental period. Twenty‐four rats were randomly assigned to four experimental groups using a computer‐generated random number sequence. Twenty‐four rats were randomly divided into four groups using a computer‐generated random number sequence: the control group, the model group, and the low‐dose and high‐dose curcumin group, with six rats in each group. In this study, the administration dose of cisplatin was based on the protocol established by Fukushima et al. (2022), and the intervention dose of curcumin referred to the protocol described by Abd El‐Kader and Taha (2020). The 0.5% CMC‐Na solution was used as the vehicle for curcumin, and saline was used for cisplatin. The treatment regimen spanned 10 consecutive days. Throughout this period, all rats received a daily oral administration. Specifically, the control and model groups were given the vehicle (0.5% CMC‐Na solution), while the low and high‐dose curcumin groups received 100 and 200 mg/kg of curcumin, respectively. On day 6 of the experiment, a single intraperitoneal injection was administered to all groups: the control group received saline, whereas the model and both curcumin treatment groups received cisplatin at 5 mg/kg. Body weight was measured and recorded every 2 days throughout the study period.

### 2.8 Biochemical Analysis of Renal Function

Under conditions designed to minimize damage to the rats, all rats were deeply anesthetized via intraperitoneal injection of sodium pentobarbital (40 mg/kg). Following confirmation of the absence of pain reflex by pinching the skin at the toe tip, the abdomen was incised to expose the aorta and kidneys. Approximately 3 mL of blood was collected from the abdominal aorta of each group of rats. Immediately after blood collection, euthanasia was performed by cervical dislocation (Xiao et al. 2025). The blood was centrifuged at 8000 rpm for 10 min to obtain serum. A part of the above‐mentioned serum was taken to measure renal biochemical parameters. Serum levels of urea nitrogen (BUN), creatinine, and CO_2_ combining power were measured using an automated biochemical analyzer (OLYMPUS AU400, Japan).

### 2.9 Renal Histopathological Assessment

After blood collection, the fresh kidneys were collected from each group of rats using surgical scissors. The residual blood was washed away with physiological saline, and the kidneys were weighed to determine the ratio of kidney weight to body weight. Subsequently, they were placed in a 10% formaldehyde solution and fixed at room temperature for 48 h. Then, the kidneys would be dehydrated, transparent, and dipped in wax. The kidneys were embedded with melted paraffin wax in specific embedding molds to form tissue wax blocks. The above‐mentioned tissue wax blocks were produced into tissue paraffin sections according to regular procedures. HE staining was performed strictly according to the steps previously reported (Lingkun et al. 2023). The renal histopathology was assessed by evaluating tubular injury (dilated or necrotic), and tubular injury was scored according to the percentage of damaged tubules (0, no injury; 1, < 25% injury; 2, 25%–50% injury; 3, 50%–75% injury; 4, > 75% injury). Similarly, the Image J software (National Institutes of Health, Bethesda, MD, USA) was used to measure renal fibrosis (Sixia et al. 2022).

### 2.10 Detection of Renal Mast Cells and Macrophages

A toluidine blue stain was performed according to the instructions in the staining kit provided by Solarbio Science & Technology Co. Ltd. (Beijing, China). Firstly, the fresh kidney paraffin sections were placed in a xylene solution. Serial proportions of the ethanol solution were washed with distilled water, and the toluidine blue staining solution was used for staining, which was then rewashed with distilled water. Mast cells (MCs) were distinguished as 95% ethanol, dehydrated as 95% ethanol, and anhydrous ethanol. The sections were blocked with xylene clear and neutral gum (Zhou, Wei, et al. 2022). Similarly, immunohistochemistry was performed according to the instructions for the staining kit provided by Servicebio Biotechnology Co Ltd. (Wuhan, China). The kidney paraffin sections were deparaffinized to water and processed in the following steps sequentially: the antigen repair was performed in 3% H_2_O_2_ solution, washed with PBS (pH 7.4) solution, 3% BSA was added dropwise to cover the tissues uniformly at room temperature, and the sections were treated with anti‐CD68 (GB11067, Servicebio Biotechnology Co Ltd., China) and incubated at 4°C overnight. The next day, the sections were washed with PBS (pH 7.4), and an HRP‐labeled secondary antibody was added. The sections were again washed with PBS (pH 7.4) and then dropped with the color‐developing solution (containing DAB solution, H_2_O_2_ solution, and phosphate buffer). After rinsing the sections with distilled water and then re‐stained with hematoxylin solution, the above sections were then dehydrated using a series of different proportions of alcohol and xylene solutions, sealed with neutral gum, and the sections were observed and photographed under a light microscope (Jindao et al. 2017).

### 2.11 LC–MS/MS Analysis

An Agilent Poroshell 120 HILIC column (4.6 × 100 mm, 2.7 μm) was used to determine the levels of urinary toxins in the serum and kidneys of rats in each group, and the column temperature was maintained at 30°C. To conduct the detection, the mass spectrometer conditions were set as follows: the dry gas temperature was set at 350°C, the flow rate was kept at 9 L/min, the nebulizer pressure was adjusted to 0.35 MPa, and the capillary voltage was maintained at 3500 V (negative ion mode). The mobile phase comprised water (0.1% acetic acid) and acetonitrile (60:40, V/V) at a 0.6 mL/min flow rate. The volume of the sample solution and standard solution was both 10 μL. It is worth noting that chromatography and mass spectrometry conditions were determined based on previous studies in our laboratory (Ma et al. 2020). Under these optimal conditions, good linearity, accuracy, and precision were obtained.

50 μL of the sample was mixed with 50 μL of the internal standard (Hippuric acid‐d5 in negative ion mode), and 100 μL of acetonitrile was added for protein precipitation. Then, the mixed sample was vortexed for 1 min to precipitate proteins and centrifuged at 10000 rpm for 10 min, and the appropriate amount of supernatant was taken for LC–MS/MS analysis.

### 2.12 Western Blotting Analysis

An appropriate amount of fresh kidney tissue was obtained with surgical scissors and placed in centrifuge tubes. The corresponding volume of RIPA lysate and some magnetic beads were added. The above‐mentioned kidney tissues were thoroughly homogenized using a tissue homogenizer and then centrifuged to collect the supernatant fraction. The above operations were performed on ice or under the condition of keeping a low temperature. The appropriate volume of the supernatant was added to the corresponding volume of SDS‐PAGE buffer and, after thorough mixing, boiled for 5 min to denature the proteins. SDS‐PAGE separated the proteins with different molecular weights and then transferred them to a methanol‐activated PVDF membrane. The PVDF membrane was sealed using 5% skimmed milk (dissolved in TBST) for 1 h at room temperature to reduce background (Shao et al. 2023). Subsequently, we incubated the above membranes with anti‐OAT3 (ThermoFisher, PA5102699), anti‐OCT2 (Novus Biologicals, NBP189417), anti‐MATE1 (ORIGENE, TA381669), anti‐MRP2 (Abcom, Ab203397), anti‐MRP4 (ORIGENE, TA327332), anti‐Kim1 (Novus Biologicals, NBP176701), anti‐NGAL (ThermoFisher, PA579591), anti‐Nrf2 (Santa Cruz Bio, sc722), anti‐Keap1 (Santa Cruz Bio, sc514914), anti‐BAX (Abcom, Ab182733), anti‐Bcl2 (Abcom, Ab194583), anti‐Cleaved caspase‐3 (Cell signaling technology, 9661), anti‐DRP1 (Abcom, Ab184247) anti‐mitofusin1 (Abcom, Ab126575), anti‐mitofusin2 (Abcom, Ab124773), anti‐Sirt3 (Santa Cruz Bio, sc365175), anti‐PGC‐1α (Santa Cruz Bio, sc518025), anti‐AMPKα (Santa Cruz Bio, sc74461), anti‐p‐AMPKα (Cell signaling technology, 50081S), and anti‐β‐actin or anti‐GAPDH and the incubation was performed on a shaker at 4°C overnight. The following day, we washed the membrane with TBST to remove excess antibody, then incubated the membrane with HRP‐conjugated antibody for 1 h at room temperature and washed with TBST to remove excess antibody to reduce background interference. We developed the above membranes using an enhanced chemiluminescence (ECL) solution and then took photographs with an automated chemiluminescence/fluorescence image analysis system (Tanon 4600 series, China). The gray values presented in the photographs were calculated and analyzed using Image J (National Institutes of Health, Bethesda, MD, USA).

### 2.13 Evaluation of Renal Oxidative Stress Levels

An appropriate amount of fresh kidney tissue from rats in each group was cut off with surgical scissors. The excess blood on the surface of the kidneys was washed away with saline, then dried with filter paper and weighed. The tissues were put into 1.5 mL centrifuge tubes containing the corresponding volume of saline and homogenized. After centrifugation at 5000 rpm for 10 min, the supernatant was collected for subsequent testing. To evaluate the degree of renal oxidative stress, the levels of renal glutathione (GSH), glutathione peroxidase (GSH‐Px), malondialdehyde (MDA), and glutathione reductase (GR) were detected according to the methods previously established (Zhang, Cui, et al. 2023). Saline was used for calibration during the experiment.

### 2.14 Detection of Mitochondrial Structure by Transmission Electron Microscopy (TEM)

According to the described protocol (Qi et al. 2020), the collected fresh kidney samples were pre‐fixed with 2.5% glutaraldehyde and re‐fixed with 1% osmium tetroxide. Then, the samples were dehydrated by different ratios of acetone in stages, infiltrated by different ratios of osmotic solution sequentially, and embedded with an Epon‐812 pure embedding agent. The sections were ultrathin sectioned at 60–90 nm using an ultrathin sectioning machine and fished onto a copper mesh. The sections were stained with uranyl acetate and lead citrate at room temperature. The JEM‐1400FLASH transmission electron microscope (Electronics Corporation (JEOL), Japan) was used for image acquisition.

### 2.15 Statistical Data Analysis

The IBM SPSS 17.0 software was used to process and analyze all the generated data from this experiment. An independent samples *t*‐test and one‐way ANOVA were also performed to analyze the obtained data. Significant differences computed in this experiment were represented as *p* < 0.05 or *p* < 0.01.

## 3 Results

### 3.1 Curcumin Alleviates Renal Injury in Cisplatin‐Induced AKI Rats

**FIGURE 1:**
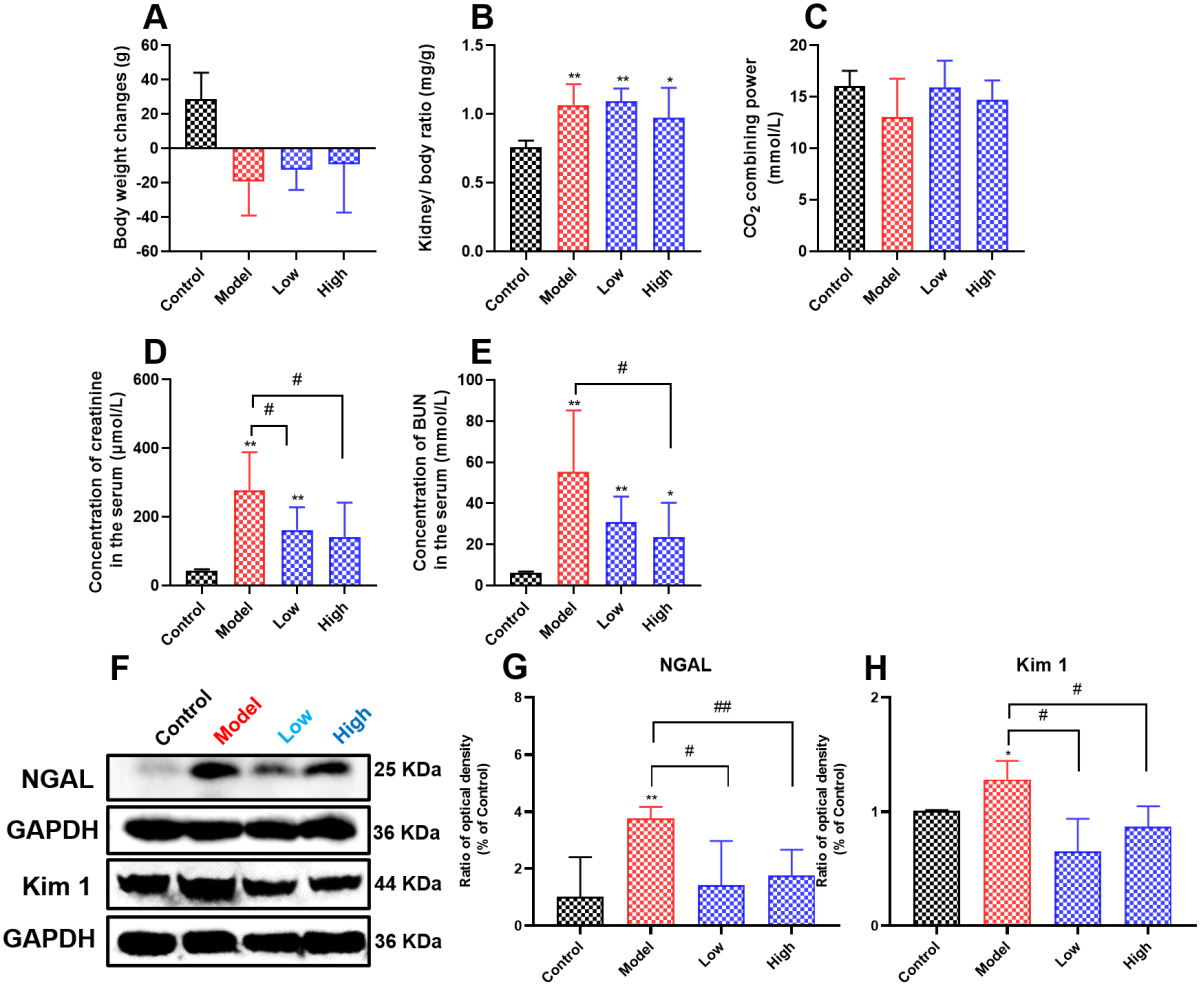
Effects of curcumin on renal function indicators in cisplatin‐induced AKI rats. (A) Body weight changes of rats in each group. (B) The ratio of the kidney to body weight of rats in each group. (C–E) Levels of serum CO_2_ combining power, creatinine, and BUN. Results are shown as mean ± standard deviation (SD) (*n* = 6). (F–H) The renal NGAL and Kim‐1 expression of rats in each group. Results are shown as mean ± SD (*n* = 3). *p < 0.05, **p < 0.01 indicate statistically significant differences compared with the control group. ^#^ *p* < 0.05, ^##^ *p* < 0.01 indicate statistically significant differences compared with the model group. Acute kidney injury (AKI); Carbon dioxide (CO_2_); Blood urea nitrogen (BUN); Neutrophil gelatinase‐associated lipocalin (NGAL); Kidney injury molecule‐1 (Kim‐1).

As shown in Figure 1A–E, we found that the administration of cisplatin resulted in a slow or even decreased body weight gain, a significant increase in the ratio of kidney to body weight (*p* < 0.01), and a decrease in the serum CO_2_ combining power, and curcumin supplementation alleviated the above phenomena, but there was no significant difference. Furthermore, the administration of cisplatin also caused a significant increase in serum biochemical parameters of renal function (creatinine and BUN) (*p* < 0.01), and curcumin supplementation significantly reduced the elevated serum creatinine and BUN levels (*p* < 0.05).

Neutrophil gelatinase‐associated lipocalin (NGAL) and kidney injury molecule 1 (KIM‐1) as novel biomarkers that are more specific for tubular injury, can be detected prior to changes in serum creatinine, and can also predict the progression and severity of AKI. As shown in Figure 1F–H, compared with the control group, we found that cisplatin administration significantly increased the expression of renal NGAL and Kim1 (*p* < 0.05). However, curcumin supplementation significantly reduced renal NGAL and Kim1 expression (*p* < 0.05). These results suggested that curcumin supplementation could alleviate renal injury caused by cisplatin.

### 3.2 Effects of Curcumin on Renal Histopathology in Cisplatin‐Induced AKI Rats

**FIGURE 2:**
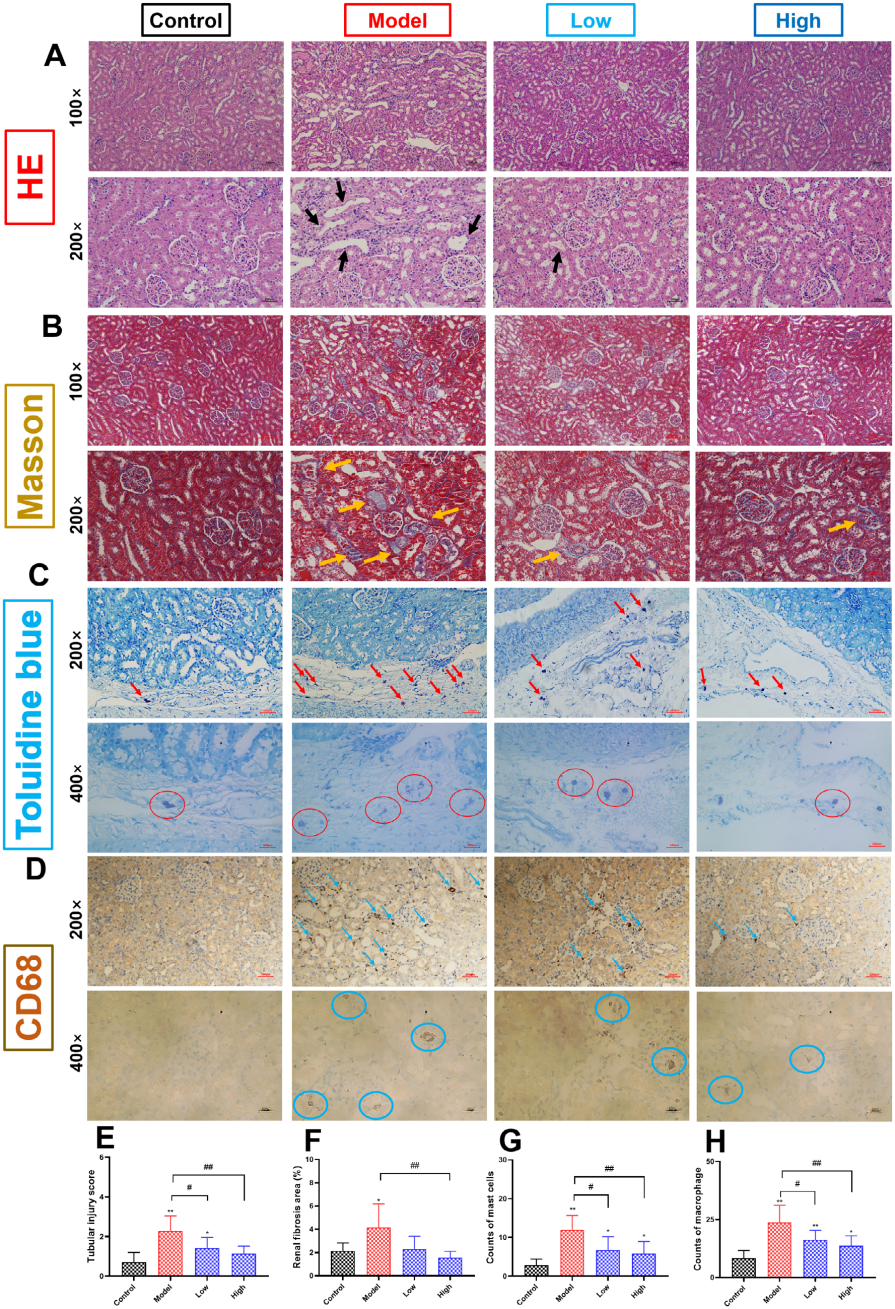
Effects of curcumin on the renal histopathology in cisplatin‐induced AKI rats. (A) HE staining of the kidneys of rats in each group (×100 and ×200). (B) Masson staining of the kidneys of rats in each group (×100 and ×200). (C) Toluidine blue staining of kidney sections of rats in each group (×200 and ×400). (D) Immunohistochemical staining of CD 68 of kidney sections of rats in each group (×200 and ×400). Black arrows indicate dilated renal tubules. Yellow arrows indicate fibrillar collagen deposits. (E) Tubular injury score of kidney sections. (F): Renal fibrosis area was measured using NIH Image J software. (G) Number of mast cells in toluidine blue staining of kidney sections. (H) Number of macrophages in immunohistochemical staining of kidney sections. Red arrows and red circles indicate mast cells. Blue arrows and blue circles indicate macrophages. Results are shown as mean ± standard deviation (SD). *p < 0.05, **p < 0.01 indicate statistically significant differences compared with the control group. ^#^ *p* < 0.05, ^##^ *p* < 0.01 indicate statistically significant differences compared with the model group. Acute kidney injury (AKI); Hematoxylin and eosin (HE) staining.

HE and Masson staining evaluated the renal histopathological changes of rats in each group. As shown in Figure 2A,B, compared with the control group, the results of HE staining showed that administration of cisplatin resulted in tubular dilatation, vacuolar degeneration of tubular epithelial cells, and infiltration of inflammatory cells. The results of Masson staining showed that administration of cisplatin led to fibrotic damage in the kidneys. As shown in Figure 2C,D, we also found that cisplatin administration triggered the recruitment of renal mast cells and macrophages. In contrast, curcumin supplementation could significantly alleviate the above‐mentioned renal histopathological damage caused by cisplatin (Figure 2E–H).

### 3.3 Effects of Curcumin on Renal Mitochondrial Structure and Dynamics in Cisplatin‐Induced AKI Rats

**FIGURE 3:**
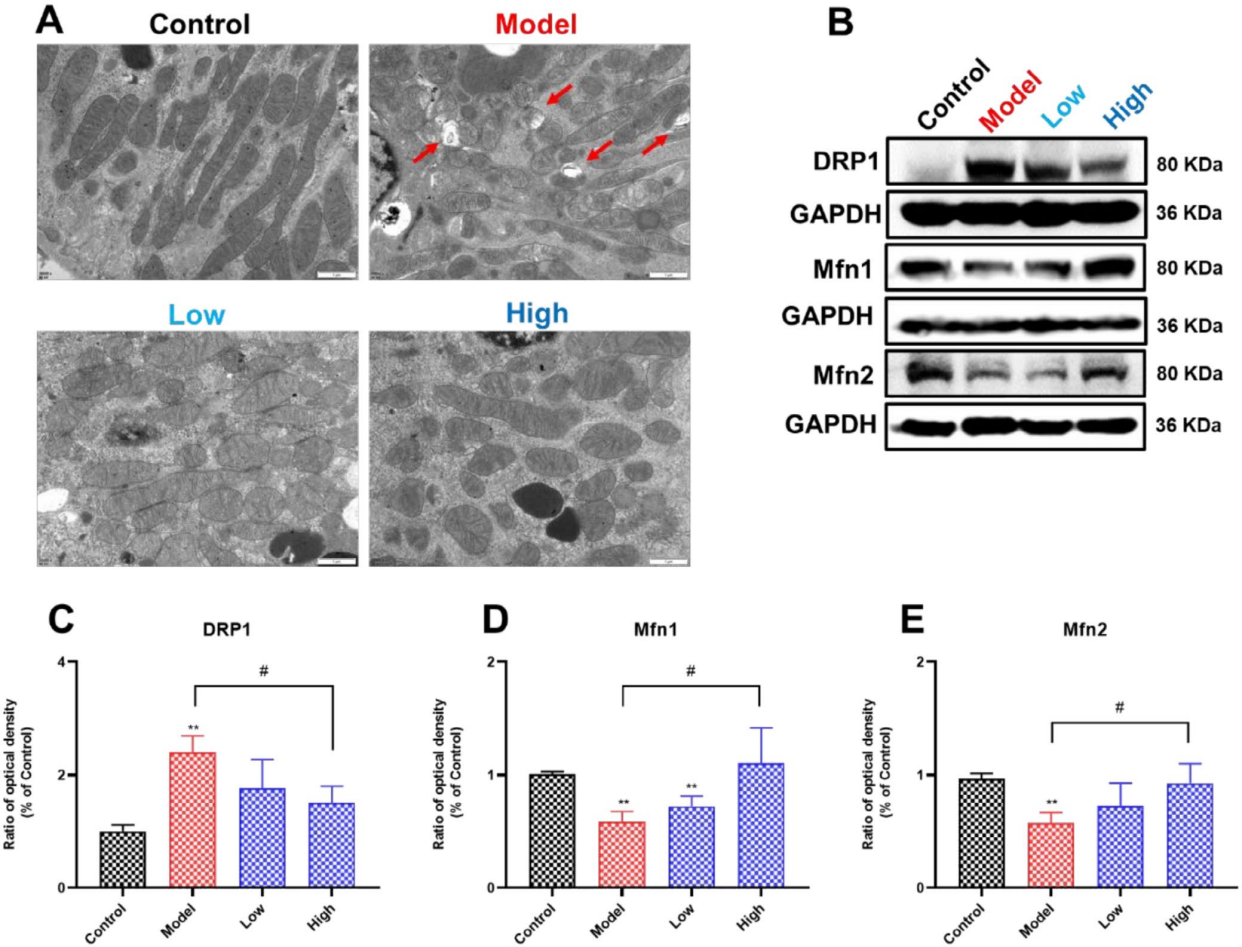
Effects of curcumin on renal mitochondrial structure and dynamics in cisplatin‐induced AKI rats. (A) The mitochondrial structure of the kidneys in each group was examined by TEM. Red arrows indicate the structurally broken mitochondria. (B–E) The expression of renal DRP1, Mfn1, and Mfn2 of rats in each group. Results are shown as mean ± standard deviation (SD) (*n* = 3). **p < 0.01 indicate statistically significant differences compared with the control group. ^#^ *p* < 0.05 indicate statistically significant differences compared with the model group. Acute kidney injury (AKI); Transmission electron microscopy (TEM); Dynamin‐related protein 1 (DRP1); Mitofusin 1 (Mfn1); Mitofusin 2 (Mfn2).

Mitochondria are the energy supply factories for cellular life activities and the important location for generating ATP from nutrients through the tricarboxylic acid cycle, electron transfer, and oxidative phosphorylation (Bock and Tait 2020). Positively charged metabolites produced by the hydrolysis of cisplatin will accumulate in negatively charged mitochondria, leading to severe vacuolization and cristae breaks of mitochondria (Jun et al. 2023). It has been reported that mitochondria are the main target of cisplatin‐induced nephrotoxicity (Zhang, Luo, et al. 2023). In this study, the morphology of renal mitochondria was observed using transmission electron microscopy (TEM). We found that curcumin significantly attenuated cisplatin‐induced mitochondrial membrane rupture, cristae breakage, swelling, and fragmentation (Figure 3A). Moreover, we also investigated renal mitochondrial dynamics. As shown in Figure 3B–E, compared with the control group, administration of cisplatin significantly increased Drp1 expression, and markedly decreased mfn1 and mfn2 expression. Notably, we found that curcumin supplementation could significantly decrease the increased Drp1 expression and increase the decreased mfn1 and mfn2 expression. These results demonstrated that curcumin supplementation could attenuate cisplatin‐induced renal injury by stabilizing mitochondrial homeostasis.

### 3.4 Effects of Curcumin on the Expression of Renal AMPK/PGC‐1α/Sirt3 Pathway in Cisplatin‐Induced AKI Rats

**FIGURE 4:**
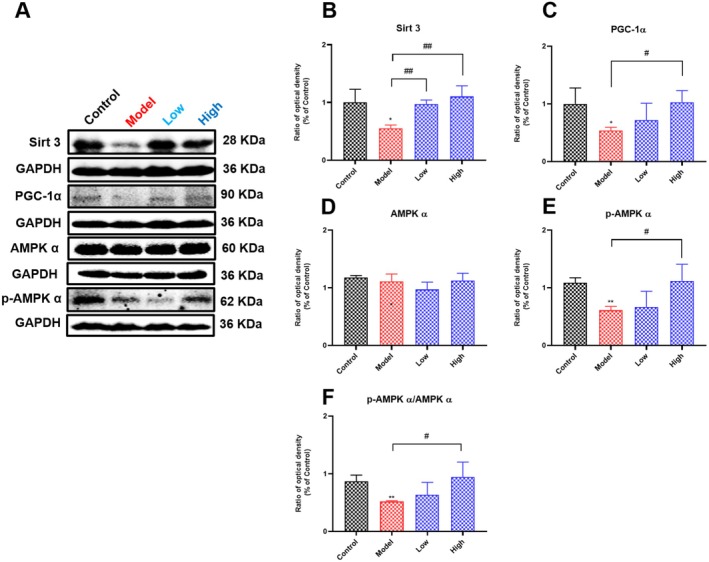
Effects of curcumin on the expression of renal AMPK‐PGC‐1α‐Sirt3 pathway in cisplatin‐induced AKI rats. (A–F) The expression of renal AMPKα, p‐AMPKα, PGC‐1α, and Sirt3 of rats in each group. Results are shown as mean ± standard deviation (SD) (*n* = 3). *p < 0.05, **p < 0.01 indicate statistically significant differences compared with the control group. ^#^ *p* < 0.05, ^##^ *p* < 0.01 indicate statistically significant differences compared with the model. Acute kidney injury (AKI); AMP‐activated protein kinase catalytic subunit alpha (AMPKα); Phosphorylated AMP‐activated protein kinase α subunit (p‐AMPKα); Peroxisome proliferator‐activated receptor‐gamma coactivator 1‐alpha (PGC‐1α); Sirtuin 3 (Sirt3).

Growing evidence has suggested that the sirtuins family regulates AKI through anti‐inflammatory, antioxidant, anti‐apoptotic, and maintenance of mitochondrial homeostasis. Of these, Sirt3 is mainly present in mitochondria. It has been reported that cisplatin administration decreases Sirt3 expression, leading to disturbed mitochondrial dynamics (Ma et al. 2020). AMPKα, a serine/threonine kinase, and a key energy‐sensing enzyme, is pivotal in maintaining cellular energy homeostasis (Daniel and Reuben J 2017). PGC‐1α, a central regulator of mitochondrial function, is extensively involved in mitochondrial biogenesis and energy metabolism (Shane and Julie 2013). Importantly, studies have established that PGC‐1α acts as a downstream target of AMPKα, while Sirt3 serves as a downstream target of PGC‐1α, mediating its effects on cellular ROS production and mitochondrial biogenesis (Priya et al. 2022; Xingxing et al. 2010). The above studies suggest that the AMPK/PGC‐1α/Sirt3 pathway plays an undeniable role in maintaining mitochondrial homeostasis. As shown in Figure 4, compared with the control group, administration of cisplatin significantly inhibited the expression of the renal AMPK/PGC‐1α/Sirt3 pathway, and curcumin supplementation could significantly upregulate the expression of the renal AMPK/PGC‐1α/Sirt3 pathway. These results indicated that curcumin supplementation could attenuate cisplatin‐induced renal injury by restoring mitochondrial dynamics through upregulating the renal AMPK/PGC‐1α/Sirt3 pathway.

### 3.5 Effects of Curcumin on Cisplatin‐Induced Mitochondrial Damage and ROS Accumulation in Renal Tubular Epithelial Cells

**FIGURE 5:**
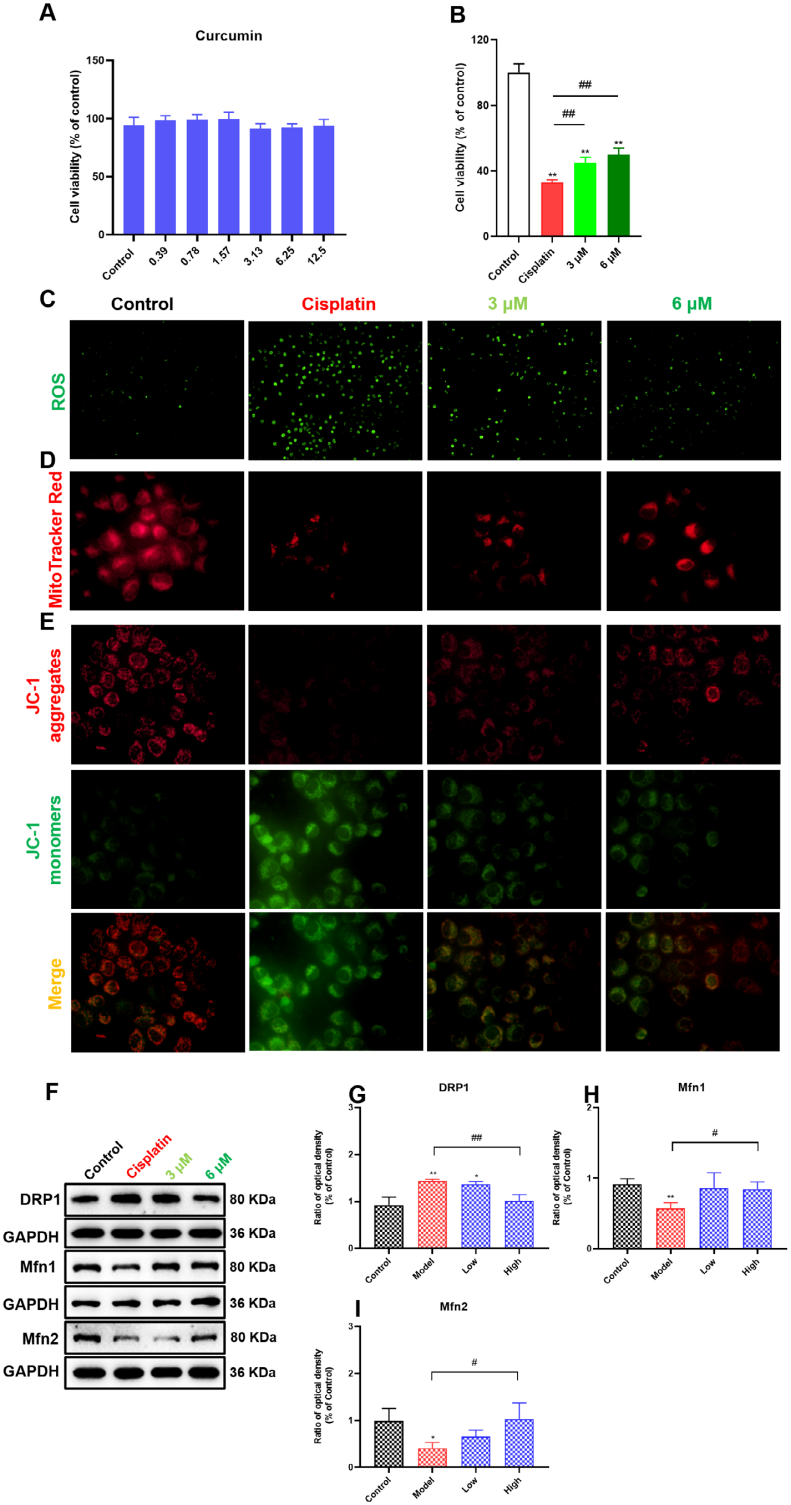
Effects of curcumin on the cytotoxicity and mitochondrial damage induced by cisplatin in renal tubular epithelial cells. (A) The viability of HK‐2 cells was determined by CCK‐8 assay after treatment with different concentrations of curcumin for 24 h. HK‐2 cells were treated with 20 μM cisplatin and curcumin (3 or 6 μM) for 24 h, and then the following parameters were measured. (B) The viability of HK‐2 cells was determined by CCK‐8 assay. (C) The ROS levels were determined by DCFH‐DA staining (100×). (D) The mitochondria of each group were examined by MitoTracker Red staining (400×). (E) The mitochondrial membrane potential was determined by JC‐1 staining (400×). (F–I) The expression of DRP1, Mfn1, and Mfn2 in each group was detected. Results are shown as mean ± standard deviation (SD) (*n* = 3). *p < 0.05, **p < 0.01 indicate statistically significant differences compared with the control group. ^#^ *p* < 0.05, ^##^ *p* < 0.01 indicate statistically significant differences compared with the model group. Human renal proximal tubular epithelial cells (HK‐2 cells); Cell Counting Kit‐8 (CCK‐8); Reactive oxygen species (ROS); Dynamin‐related protein 1 (DRP1); Mitofusin 1 (Mfn1); Mitofusin 2 (Mfn2).

As shown in Figure 5A,B, we used different concentrations of curcumin to intervene in HK‐2 cells for 24 h to investigate curcumin's toxicity. The results showed that even 12.5 μM curcumin did not cause significant renal tubular epithelial cell damage, and curcumin (3 or 6 μM) could significantly alleviate renal tubular epithelial cell damage caused by cisplatin (*p* < 0.01). Furthermore, we also found that curcumin significantly alleviated mitochondrial damage (including the decrease in mitochondrial membrane potential) and the accumulation of ROS caused by cisplatin (Figure 5C–E). Notably, curcumin could stabilize mitochondrial dynamics and alleviate cisplatin‐induced mitochondrial damage by reducing the expression of DRP1 and elevating the expression of Mfn1 and Mfn2 (Figure 5F–I). These results suggested that curcumin could alleviate cisplatin‐induced renal tubular epithelial cell damage by reducing mitochondrial damage and ROS accumulation.

### 3.6 Effects of Curcumin on the Levels of Renal Oxidative Stress and Apoptosis in Cisplatin‐Induced AKI Rats

**FIGURE 6:**
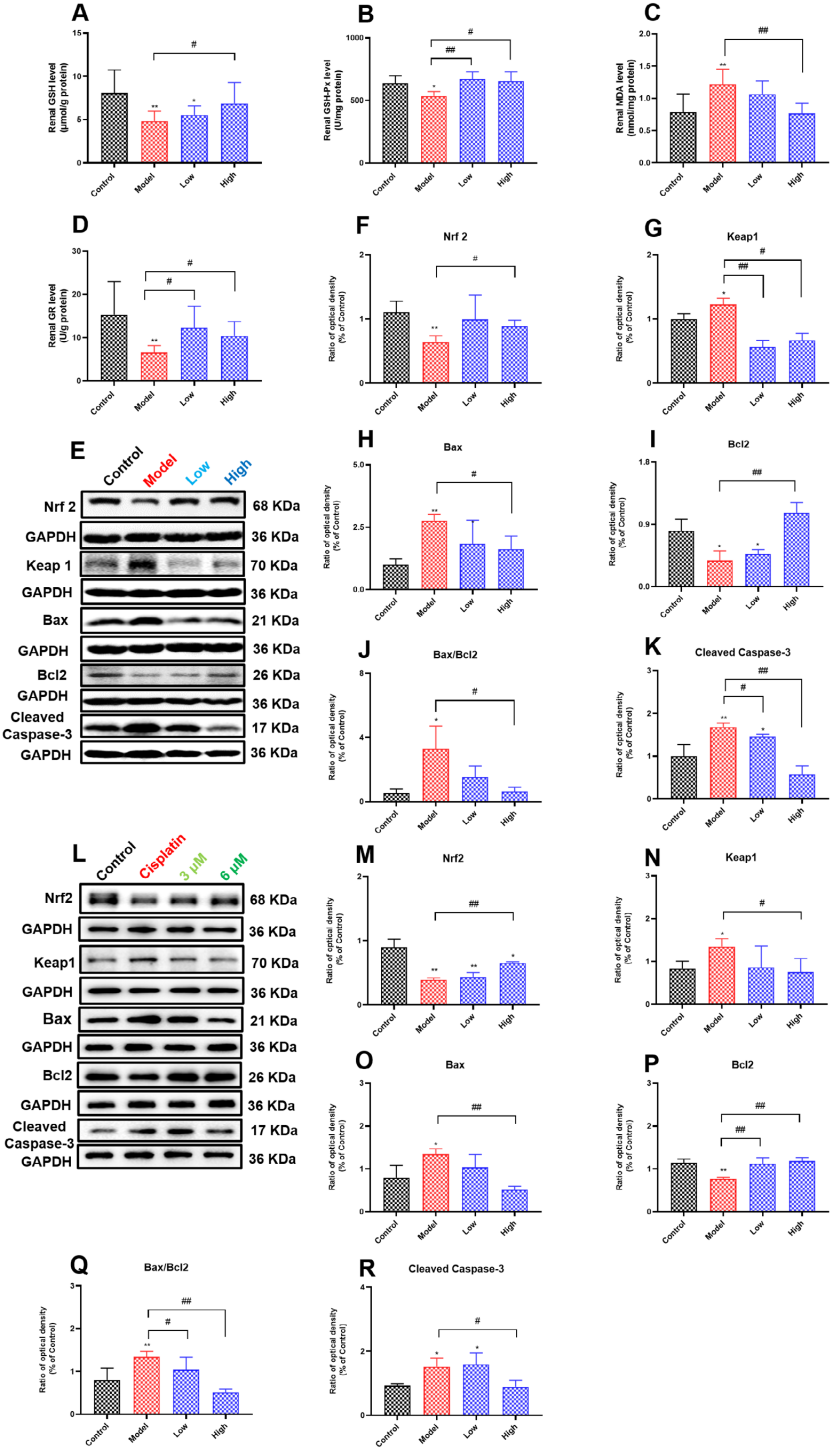
Effects of curcumin on the levels of renal oxidative stress and apoptosis in cisplatin‐induced AKI rats. (A–D) The levels of renal GSH, GSH‐Px, MDA, and GR of rats in each group. Results are shown as mean ± standard deviation (SD) (*n* = 6). (E–K) The expression of renal Nrf2, Keap1, Bax, Bcl2, and Cleaved Caspase‐3 of rats in each group. (L–R) HK‐2 cells were treated with 20 μM cisplatin and curcumin (3 or 6 μM) for 24 h, and then the expression of Nrf2, Keap1, Bax, Bcl2, and Cleaved Caspase‐3 in each group was detected. Results are shown as mean ± standard deviation (SD). *p < 0.05, **p < 0.01 indicate statistically significant differences compared with the control group. ^#^ *p* < 0.05, ^##^ *p* < 0.01 indicate statistically significant differences compared with the model group. Acute kidney injury (AKI); Reduced glutathione (GSH); Glutathione peroxidase (GSH‐Px); malondialdehyde (MDA); glutathione reductase (GR); renal nuclear factor erythroid 2‐related factor 2 (Nrf2); Kelch‐like ECH‐associated protein 1 (Keap1); Bcl2‐associated X protein (Bax); B‐cell lymphoma 2 (Bcl2); Human renal proximal tubular epithelial cells (HK‐2 cells).

The nephrotoxicity induced by cisplatin involves a variety of pathological mechanisms, including oxidative/nitrosative stress. Following entry into renal tubular cells, cisplatin could degrade or inactivate the sulfhydryl‐containing antioxidants glutathione and metallothionein and affect the activity of antioxidant enzymes, leading to elevated ROS levels (Chun‐Yan et al. 2021). Simultaneously, the body is also equipped with antioxidant defense systems: non‐enzymatic systems (e.g., glutathione (GSH)) and enzymatic systems (e.g., glutathione peroxidase (GSH‐Px) and glutathione reductase (GR)), which protect the cells from oxidative stress. Moreover, malondialdehyde (MDA) is a biomarker of oxidative stress in the body and oxidative damage to lipids (Antonio et al. 2022). As shown in Figure 6A–D, compared with the control group, cisplatin administration significantly decreased renal GSH, GSH‐Px, and GR levels and markedly increased renal MDA levels. Notably, we found that curcumin supplementation could significantly increase the reduced renal GSH, GSH‐Px, and GR levels and also reduce the increased renal MDA level.

The transcription factor Nrf2 is a major regulator of the antioxidant response and has attracted attention to the oxidative stress and apoptosis induced by cisplatin (Mapuskar et al. 2023). As shown in Figure 6E–K, compared with the control group, cisplatin administration significantly decreased renal Nrf2 and Bcl2 expression and markedly increased Keap1, Bax, and Cleaved Caspase‐3 expression. However, curcumin supplementation could considerably increase renal Nrf2 and Bcl2 expression and reduce Keap1, Bax, and Cleaved Caspase‐3 expression. Similarly, in vitro experiments also found similar results that curcumin effectively modulated the Nrf2/Keap1 and Bax/Bcl2/Caspase‐3 pathways and attenuated cisplatin‐induced oxidative stress and apoptosis in the kidney (Figure 6L–R). These results suggest that curcumin supplementation effectively modulates the Nrf2/Keap1 and Bax/Bcl2/Caspase‐3 pathways, attenuating cisplatin‐induced oxidative stress and apoptosis in the kidney.

### 3.7 Effects of Curcumin on the Level of Serum and Renal Urinary Toxins in Cisplatin‐Induced AKI Rats

**FIGURE 7:**
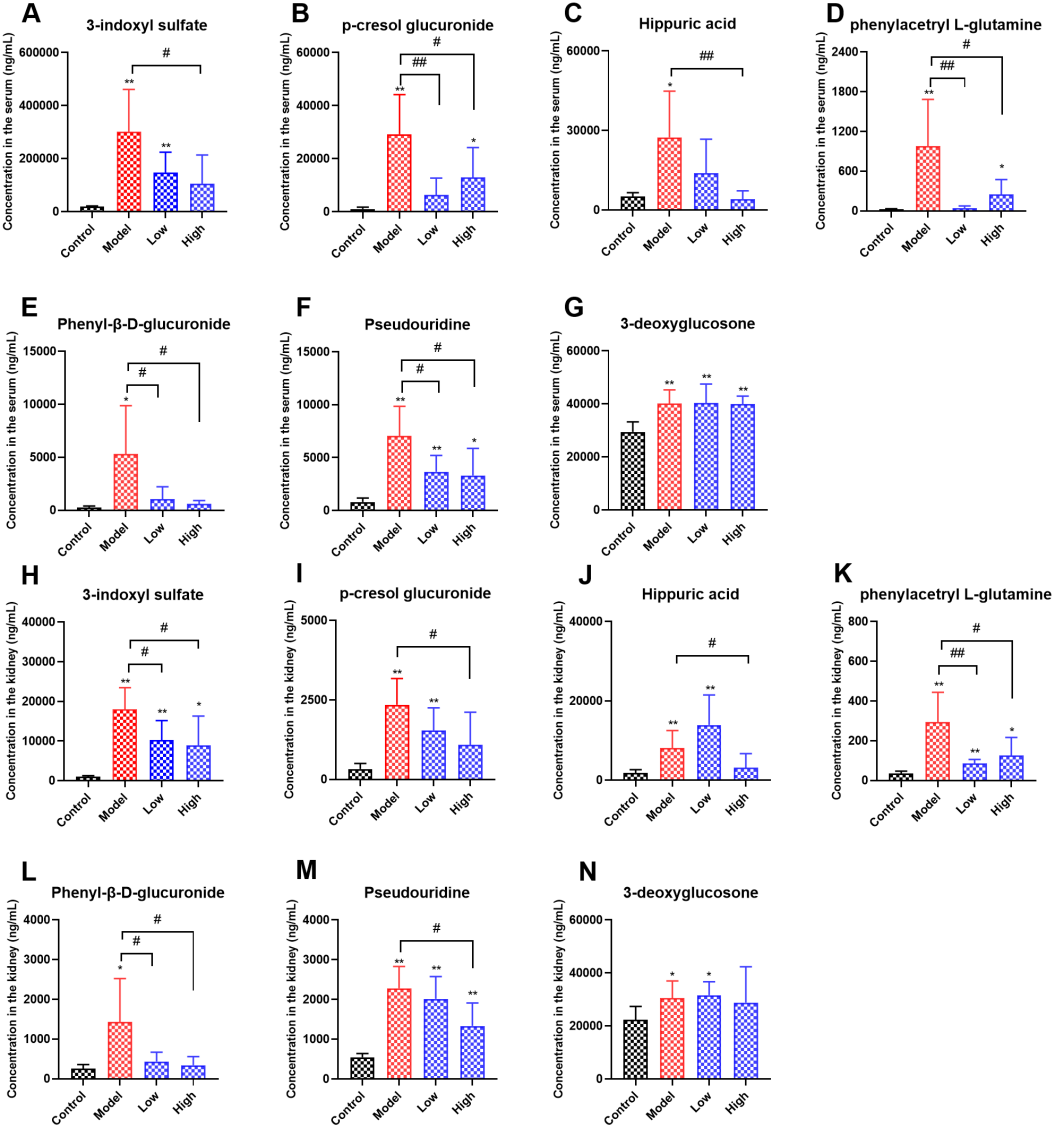
Effects of curcumin on serum and renal urinary toxins levels in cisplatin‐induced AKI rats. (A–G) Serum 3‐indoxyl sulfate, *p*‐cresol glucuronide, Hippuric acid, phenylacetryl L‐glutamine, Phenyl‐β‐D‐glucuronide, Pseudouridine, and 3‐deoxyglucosone levels in each group of rats. (H–N) Renal 3‐indoxyl sulfate, *p*‐cresol glucuronide, Hippuric acid, phenylacetryl L‐glutamine, Phenyl‐β‐D‐glucuronide, Pseudouridine, and 3‐deoxyglucosone levels in each group of rats. Results are shown as mean ± standard deviation (SD) (*n* = 6). *p < 0.05, **p < 0.01 indicate statistically significant differences compared with the control group. ^#^ *p* < 0.05, ^##^ *p* < 0.01 indicate statistically significant differences compared with the model group. Acute kidney injury (AKI).

It has been reported that urinary toxins and their metabolites can affect mitochondrial complex III and IV enzyme activities and activate NAD (P)H oxidase in various cell types to induce oxidative stress, which negatively affects mitochondrial energy and directly triggers apoptosis and necrosis in renal tubular cells (Trace et al. 2019). Notably, the typical clinical feature of AKI is a dramatic loss of renal function in a short period (Wang et al. 2024). Urinary toxins are usually excreted by the kidneys. Unfortunately, these toxins accumulate in the patient's body (especially in the plasma) due to the reduced ability of their secretion, leading to electrolyte disturbances and affecting the functioning of other organs (Ma et al. 2020). As shown in Figure 7, compared to the control group, we found that the administration of cisplatin induced a significant increase in serum and renal 3‐indoxyl sulfate, *p*‐cresol glucuronide, Hippuric acid, phenylacetryl L‐glutamine, Phenyl‐β‐D‐glucuronide, Pseudouridine, and 3‐deoxyglucosone levels (*p* < 0.05). However, curcumin supplementation could significantly reduce these urinary toxins (except 3‐deoxyglucosone) levels in serum and kidneys (*p* < 0.05). These results demonstrated that curcumin supplementation could alleviate cisplatin‐induced renal injury by decreasing serum and renal urinary toxins levels.

### 3.8 Effects of Curcumin on the Expression of Renal Transporters in Cisplatin‐Induced AKI Rats

**FIGURE 8:**
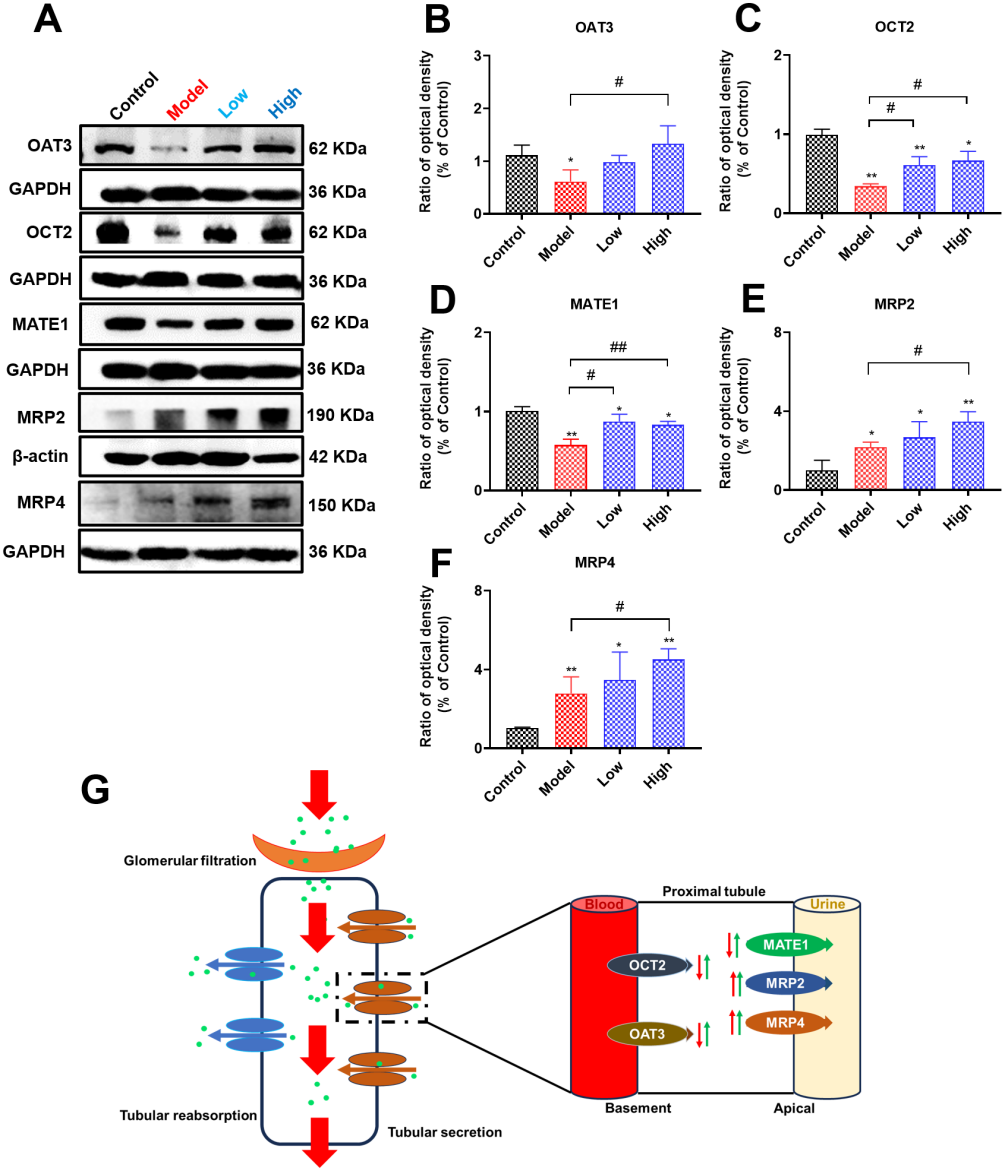
Effects of curcumin on the expression of renal OAT3, OCT2, MATE1, and MRP2/4 in cisplatin‐induced AKI rats. (A–F) The expression of renal OAT3, OCT2, MATE1, and MRP2/4 in rats of each group. (G) Schematic representation of the changes in renal transporters. Results are shown as mean ± standard deviation (SD) (*n* = 3). *p < 0.05, **p < 0.01 indicate statistically significant differences compared with the control group. ^#^ *p* < 0.05, ^##^ *p* < 0.01 indicate statistically significant differences compared with the model group. Organic anion transporter 3 (OAT3); Organic cation transporter 2 (OCT2); Multidrug and toxin extrusion protein 1 (MATE1); Multidrug resistance–associated protein 2 (MRP2); Multidrug resistance–associated protein 4 (MRP4); Acute kidney injury (AKI).

The kidneys eliminate drugs or metabolic wastes from the body through glomerular filtration, tubular secretion, and tubular reabsorption (Yanrong et al. 2024). In order to perform secretory or reabsorption functions, the kidney is particularly rich in mitochondria. However, insufficient energy supply can influence renal secretory or reabsorption functions and result in the accumulation of urinary toxins and kidney injury (Zhibo et al. 2020). In renal tubular epithelial cells, the uptake transporters on the basolateral membrane and the efflux transporters on the apical membrane are mainly responsible for the secretory or reabsorption function of the renal tubule (Yanrong et al. 2023). Because of the dramatic short‐term loss of renal function and accumulation of urinary toxins associated with AKI, we investigated the renal transporter expression. As shown in Figure 8, compared with the control group, cisplatin administration significantly decreased the expression of renal OAT3, OCT2, and MATE1 and markedly increased renal MRP2 and MRP4 expression. Furthermore, we found that curcumin supplementation could significantly increase the reduced OAT3, OCT2, and MATE1 expression and increase renal MRP2 and MRP4 expression considerably. These results demonstrated that curcumin supplementation could effectively regulate the expression of renal transporters, thereby attenuating cisplatin‐induced renal injury.

## 4 Discussion

The nephrotoxicity of cisplatin is cumulative and dose‐dependent, usually requiring dose reduction or discontinuation (Chun‐Yan et al. 2021). It has been reported that approximately 30% of patients who initiate the administration of cisplatin will suffer from AKI, which is characterized by rapid loss of renal function. Notably, some patients will experience renal tubulopathy, chronic inflammation, capillary thinning, tubulointerstitial fibrosis, and glomerulosclerosis, leading to progression to CKD (Siyao et al. 2023). These factors lead to the necessary suspension or discontinuation of cisplatin administration in many patients to avoid renal damage. Considering there are still no effective drugs for the treatment of AKI in the clinic (Ling et al. 2021), there is an urgent need for novel therapeutic approaches that can effectively mitigate AKI. Many medicinal plants are gradually attracting researchers' attention as therapeutic agents (Radwan et al. 2023). Our group has been devoted to screening active ingredients from traditional Chinese medicine for their nephroprotective effects. Of these, curcumin, a polyphenolic compound in the turmeric plant with antioxidant, anti‐inflammatory, antimicrobial, antimutagenic, and anticarcinogenic activities and low toxicity, is highly anticipated (Ivo F et al. 2023).

In this study, our results found that curcumin could significantly alleviate the abnormal renal biochemical indices and histopathological damage (e.g., vacuolar degeneration of renal tubular epithelial cells and tubular dilatation). Furthermore, we also found that curcumin supplementation could reduce the increased biomarkers of tubular injury (e.g., NGAL and Kim 1) induced by cisplatin. These results suggested that curcumin supplementation could attenuate cisplatin‐induced renal injury and serve as a new strategy for preventing AKI. Curcumin has been reported to ameliorate renal injury by ameliorating mitochondrial electron transport chain (ETC) dysregulation, redox dysregulation, and primarily by maintaining mitochondrial homeostasis (Ling et al. 2021). Previous studies have suggested that mitochondria are the main target of cisplatin‐induced nephrotoxicity (Motwani et al. 2022). Therefore, further investigation needs to investigate whether curcumin alleviates cisplatin‐induced nephrotoxicity by ameliorating mitochondrial damage.

The kidney is a mitochondria‐rich and high‐energy organ and requires a proper supply of mitochondria to maintain normal kidney function (Zou et al. 2021). Mitochondria are the main target of cisplatin‐induced nephrotoxicity (Zhang, Cui, et al. 2023). The positively charged metabolites produced by cisplatin hydrolysis accumulate in negatively charged mitochondria and cause structural or functional damage to mitochondria, especially in renal proximal tubule cells with high mitochondrial density (Jun et al. 2023). Furthermore, cisplatin‐induced mitochondrial damage also increases the production of ROS, which triggers oxidative stress and accelerates mitochondrial damage (Zhou, Dai, et al. 2022). Mitochondrial dysfunction has been proposed as a promising target for the treatment of AKI (Ling et al. 2021). In this study, we found that administration of cisplatin resulted in altered mitochondrial dynamics, affecting the dynamic balance of mitochondria and damaging the mitochondrial structure, which led to renal injury. It is worth noting that Sirt3, which is predominantly found in mitochondria, protects the kidney by improving mitochondrial dynamics and facilitating energy cycling, reducing oxidative stress and inflammatory responses (Huang et al. 2022). Similar to Li et al. (Ma et al. 2020), our study also found that cisplatin administration decreased Sirt3 expression, which disturbed mitochondrial dynamics.

AMPKα, a serine/threonine kinase, and a key energy‐sensing enzyme, is pivotal in maintaining cellular energy homeostasis. Its activation is primarily driven by the phosphorylation of the Thr172 residue on the α subunit, a process regulated by AMP/ADP levels (Daniel and Reuben J 2017). PGC‐1α, a central regulator of mitochondrial function, is extensively involved in mitochondrial biogenesis and energy metabolism. As a transcriptional coactivator, PGC‐1α does not directly bind to DNA but instead interacts with transcription factors to modulate the expression of genes related to metabolism, thereby regulating cellular energy balance and adaptive metabolic responses (Shane and Julie 2013). Importantly, studies have established that PGC‐1α acts as a downstream target of AMPKα, while Sirt3 serves as a downstream target of PGC‐1α, mediating its effects on cellular ROS production and mitochondrial biogenesis (Priya et al. 2022; Xingxing et al. 2010). Emerging evidence highlights the therapeutic potential of curcumin in diseases characterized by mitochondrial dysfunction, primarily through its ability to enhance mitochondrial structure and function. For instance, Cheng et al. demonstrated that curcumin mitigates pulmonary fibrosis by modulating extracellular matrix remodeling and mitochondrial function (Meng‐Hsuan et al. 2024). Similarly, Hou et al. reported that curcumin improves mitochondrial quality and alleviates sepsis‐induced cardiac dysfunction by reducing mitochondrial fission and promoting mitochondrial biogenesis (Dongyao et al. 2024). In this study, we found that curcumin supplementation modulates mitochondrial dynamics by activating the AMPK/PGC‐1α/Sirt3 pathway, thereby attenuating cisplatin‐induced renal injury. Similar to our study, N‐acetylcysteine protects against renal ischemia–reperfusion injury by maintaining mitochondrial homeostasis via the AMPK/PGC‐1α/Sirt3 axis (Peerapanyasut et al. 2020). These results suggest that cisplatin‐induced nephrotoxicity stems from mitochondrial damage, and curcumin alleviates renal injury by restoring mitochondrial homeostasis through the AMPK/PGC‐1α/Sirt3 pathway.

Cisplatin‐induced nephrotoxicity involves a variety of pathological mechanisms, including oxidative/nitrosative stress, mitochondrial dysfunction, inflammation, and cell cycle dysregulation (Lin et al. 2024). Following entry into renal tubular cells, cisplatin could degrade or inactivate the sulfhydryl‐containing antioxidants glutathione and metallothionein, as well as affect the activity of antioxidant enzymes, leading to elevated levels of ROS (Chun‐Yan et al. 2021). Additionally, cisplatin can trigger a series of mitochondria‐related events, including structural changes, increased permeability, and decreased membrane potential, leading to the release of Cytochrome C and programmed cell death (Linlin et al. 2013). Our results found that curcumin significantly alleviated mitochondrial damage (including the decrease in mitochondrial membrane potential) and the accumulation of ROS caused by cisplatin. Increasing evidence suggested that curcumin could scavenge residual ROS, regulate various signal transduction pathways involved in apoptosis (Hu et al. 2023; Ramadan et al. 2024), and down‐regulate NF‐κB to inhibit inflammation (Asal Jalal et al. 2021).

Moreover, Nrf2 may be a potential target of curcumin (Ling et al. 2021), and curcumin exhibits its antioxidant potential through activation of the AMPK/Nrf2/ARE/Keap1 pathway (Hu et al. 2023). In this study, curcumin supplementation was found to reverse the cisplatin‐induced decrease in GSH levels and activity of antioxidant enzymes (e.g., GSH‐PX and GR) and to reduce the level of MDA (a marker of lipid peroxidation) to alleviate renal oxidative stress injury caused by cisplatin. Curcumin supplementation also alleviated the renal apoptosis induced by cisplatin by modulating Keap1/Nrf2 and Bcl‐2/Bax/Caspase‐3 pathways. Similar to our results, Wu et al. reported that curcumin inhibited oxidative stress through the Nrf2/HO‐1 pathway, ameliorated apoptosis by activating the PI3K/Akt pathway, and thus alleviated rhabdomyolysis‐induced AKI (Jindao et al. 2017). Li et al. also reported that curcumin effectively ameliorated ischemia/reperfusion‐induced renal tubular apoptosis or necrosis, reduced the levels of oxidative stress markers, and increased the levels of antioxidant proteins (Nrf2, HO‐1, and SOD2) in both in vitro and in vivo experiments (Ling et al. 2021).

It has been reported that urinary toxins and their metabolites negatively affect mitochondrial energetics, resulting in reduced energy transfer, impaired mitochondrial complex III and IV enzyme activity, and increased oxidant production (Trace et al. 2019). Urinary toxins accumulate in the blood and kidneys, leading to metabolic disorders and aggravated kidney injury (André et al. 2023). Renal replacement therapy, such as hemodialysis, can only partially restore renal function by removing smaller amounts of unbound solutes while leaving more considerable amounts of protein‐bound urinary toxins (Faria and de Pinho 2021). Indoxyl sulfate, a protein‐bound urinary toxin, induces oxidative stress by activating NAD (P)H oxidase in various cell types (Mikito et al. 2015), directly triggers apoptosis and necrosis in renal tubular cells, and reduces antioxidant capacity (Cheng et al. 2020). In this study, we found that the nephrotoxicity caused by cisplatin resulted in significantly elevated serum and renal levels of urinary toxins (e.g., indoxyl sulfate) and that curcumin supplementation could alleviate renal injury by reducing the elevated levels of urinary toxins.

The kidney is mainly responsible for regulating various physiological functions, such as removing metabolic wastes and toxins and maintaining electrolyte and fluid balance (Meyer‐Schwesinger et al. 2022). The kidney performs the movement of solutes to (reabsorption) or (secretion) peritubular capillaries. To ensure an adequate supply of ATP, the kidney is particularly rich in mitochondria. Inadequate energy supply (e.g., mitochondrial damage due to cisplatin) leads to urinary toxin accumulation and kidney injury (Zhibo et al. 2020). Cisplatin is mainly excreted through the kidney and accumulated in the proximal tubules of the kidney (Davoudi et al. 2022). The nephrotoxicity of cisplatin primarily influences renal tubular cells and leads to oxidative stress‐mediated cell death and dysfunction (Casanova et al. 2020). The role of transporters in drug absorption, distribution, clearance, and elimination is now widely recognized (Varma 2023). There are two main classes of transporters in the proximal renal tubule: solute carrier (SLC), located on the basolateral membrane, which is mainly responsible for uptake, and ATP‐binding cassette (ABC) transporters, located on the brush border, which are mainly responsible for efflux (Łapczuk‐Romańska et al. 2023). In this study, we found that the administration of cisplatin could affect the expression of transporters, leading to the accumulation of urinary toxins in the serum or kidney and aggravating renal injury. In contrast, curcumin supplementation could promote the expression of both uptake and efflux transporters, reduce the accumulation of urinary toxins, and alleviate renal injury. Similar to our results, Yuan et al. reported that hyperoside could protect against cisplatin‐induced AKI by upregulating the expression and function of renal OAT1 and promoting the excretion of indole sulfate (Yuan et al. 2023). Liu et al. also revealed that JBP485 ameliorated cisplatin‐induced renal injury by upregulating renal MRP2 expression and promoting urinary toxin (e.g., indole sulfate) excretion (Liu et al. 2012). This study suggests that curcumin supplementation shows promise in alleviating cisplatin‐induced nephrotoxicity and could offer clinical benefits to patients receiving cisplatin chemotherapy. However, further clinical trials are needed to confirm these preclinical results and to establish an optimal dosing regimen.

## 5 Conclusions

In summary, cisplatin‐induced nephrotoxicity was caused by dysregulation of mitochondrial homeostasis, leading to excessive oxidative stress and apoptosis in the kidney, and dysregulation of renal transporters' expression, leading to accumulation of urinary toxins. However, curcumin could restore mitochondrial homeostasis through the AMPK/PGC‐1α/Sirt3 pathway, alleviate renal oxidative stress and apoptosis through the Nrf2/Keap1 and Bax/Bcl2/Caspase‐3 pathways, and regulate renal transporters' expression to alleviate urinary toxins accumulation, which ultimately alleviated cisplatin‐induced nephrotoxicity.

## Author Contributions

**Xin'an Wu:** data curation (equal), formal analysis (equal), funding acquisition (equal), investigation (equal), methodology (equal), project administration (equal), supervision (equal), writing – review and editing (equal). **Mingkang Zhang:** data curation (equal), investigation (equal), methodology (equal), project administration (equal), supervision (equal), writing – original draft (equal), writing – review and editing (equal). **Yazhi Wang:** investigation (equal), methodology (equal), software (equal), supervision (equal), writing – original draft (equal), writing – review and editing (equal). **Xiujuan Wang:** formal analysis (equal), investigation (equal), methodology (equal), software (equal), writing – review and editing (equal). **Yan Zhou:** formal analysis (equal), investigation (equal), methodology (equal), software (equal), supervision (equal), writing – review and editing (equal). **Yanrong Ma:** data curation (equal), investigation (equal), methodology (equal), software (equal), supervision (equal), writing – review and editing (equal).

## Conflicts of Interest

The authors declare no conflicts of interest.

## Data Availability

Upon request, the data used in this paper can be provided.
